# Targeting of activated fibroblasts for imaging and therapy

**DOI:** 10.1186/s41181-019-0069-0

**Published:** 2019-07-25

**Authors:** Thomas Lindner, Anastasia Loktev, Frederik Giesel, Clemens Kratochwil, Annette Altmann, Uwe Haberkorn

**Affiliations:** 1 Department of Nuclear Medicine, University Hospital Heidelberg, Im Neuenheimer Feld 400, 69120 Heidelberg, Germany; 2 Clinical Cooperation Unit Nuclear Medicine, German Cancer Research Center (DKFZ), Heidelberg, Germany; 3 Translational Lung Research Center Heidelberg (TLRC), German Center for Lung Research (DZL), Heidelberg, Germany

**Keywords:** Cancer-associated fibroblast, Tumor stroma, Fibroblast activation protein, Inhibitors, Imaging

## Abstract

Tumors form a complex environment consisting of a variety of non-malignant cells. Especially cancer-associated fibroblasts have been shown to have an important role for different aspects of malignant tumors such as migration, metastasis, resistance to chemotherapy and immunosuppression. Therefore, a targeting of these cells may be useful for both imaging and therapy. In this respect, an interesting target is the fibroblast activation protein (FAP) which is expressed in activated fibroblasts, but not in quiescent fibroblasts, giving the opportunity to use this membrane-anchored enzyme as a target for radionuclide-based approaches for diagnosis and treatment of tumors and for the diagnosis of non-malignant disease associated with a remodelling of the extracellular matrix.

## Introduction

Tumors can be considered as an assembly not only of malignant cells, but also of stroma cells which include vascular cells, inflammatory cells and fibroblasts. These are subsumed under the term stroma. The stroma may represent > 90% of the tumor mass in tumors with desmoplastic reaction such as breast, colon and pancreatic carcinoma. Especially a subpopulation of fibroblasts called cancer-associated fibroblasts (CAFs) is known to be involved in growth, migration and progression of the tumor. CAFs have a heterogeneous origin, they may develop from local fibroblasts, circulating fibroblasts, vascular endothelial cells via endothelial to mesenchymal transition, adipocytes, bone marrow derived stem cells or even from cancer cells via endothelial to mesenchymal transition (Cirri and Chiarugi 2011; Garin-Chesa et al. 1990; Gascard and Tlsty 2017; Ischii et al. 2016; Kalluri 2016; Öhlund et al. 2014). This heterogeneity of origin leads to a heterogeneous proteome with different functionality. In general, activation of these cells leads to changes in morphology with a more stellate shaped as opposed to a spindle shaped form. Furthermore, the activated fibroblasts are able to migrate, proliferate, production of extracellular matrix, express different markers such as α smooth muscle actin (αSMA), platelet derived growth factor β (PDGFR β) and fibroblast activation protein (FAP) whereas normal fibroblasts express fibroblast stimulating protein 1 (FSP1) and α1β1 integrin (Kalluri 2016). Activated fibroblast occur not only in tumors, but also in healing wounds and diseases with matrix remodelling such as chronic inflammation, heart infarction and liver and lung fibrosis (Aimes et al, 2003; Bauer et al. 2006; Egger et al. 2017; Hamson et al. 2014; Nagaraju et al. 2017; Tillmanns et al. 2015; Uitte de Willige et al. 2013).

## Interaction of CAFs with tumor cells

CAFs interact with the tumor cells in many ways. The remodelling of the extracellular matrix for example by collagenolysis promotes the invasion of tumor cells and may be involved in the induction of epithelial to mesenchymal transition (Chen and Kelly 2003; Fuyuhiro et al. 2012; Gao et al. 2010; Goodman et al. 2003; Kim et al. 2015). Furthermore, the changes in the microenvironment including an increase in stiffness of the tissue may be involved in therapy resistance as well as immunosuppression (Attieh and Vignjevic 2016; Erez et al. 2010; Kraman et al. 2010; Turley et al. 2015).

Especially the activity of the fibroblast activation protein (FAP) seems to be involved in the angiogenesis via the cleavage products of its substrates (Hamson et al. 2014; Keane et al. 2011; Zukowska et al. 2003). This is substantiated by studies showing a correlation between FAP expression and microvessel density in tumors (Keane et al. 2011; Huang et al. 2004). FAP expression has also been found to be associated with a poor prognosis in a variety of tumors such as colon, pancreatic, ovarian and hepatocellular carcinoma (Cohen et al. 2008; Henry et al. 2007; Ju et al. 2009; Zhang et al. 2011).

Besides these activities CAFs also secrete a variety of molecules, mostly growth factors and cytokines, which may induce epithelial to mesenchymal transition. The best described factor up to now is the transforming growth factor β (TGFβ). The overexpression of TGFβ has been shown to induce an increased expression of a variety of proteins known as mesenchymal markers including fibronectin, vimentin and matrix metalloproteinase (Yu et al. 2014; Erdogan and Webb 2017). Other factors excreted by stromal cells are hepatocyte growth factor (HGF), fibroblast growth factor (FGF) 1 and 2, stromal cell derived factor 1(SDF1/CXCL12), chemokine (C-C motif) ligands (CCL) 2, 5,7 and 16, interleukin 6 and 8 and platelet derived growth factor (Attieh and Vignjevic 2016; Erdogan and Webb 2017).

Furthermore, metabolic cooperation between CAFs and tumor cells occurs. This is realized by an increased expression of enzymes related to aerobic glycolysis such as lactate dehydrogenase and M2 pyruvate kinase (Guido et al. 2012; Martinez-Outschoorn et al. 2012; Öhlund et al. 2014; Pavlides et al. 2009) which has been named reverse Warburg effect. This results in the secretion of metabolites such as lactate and ketones which are taken up by tumor cells via monocarboxylate transporters (MCTs) to enter the oxidative mitochondrial pathway. However, there may be also other metabolic patterns with high expression of MCTs and low expression of glucose transporters in activated fibroblasts (Koukourakis et al. 2006). Therefore, diverse metabolic patterns are possible in different tumor types which have been identified by Choi et al. (2013) as Warburg type (glycolysis in tumor cells, non-glycolysis in stroma cells), reverse Warburg type (non-glycolysis in tumor cells, glycolysis in stroma cells), mixed type (glycolysis in both cell types) and null type with no glycolysis in both cell types. Altogether this puts into question whether the signal obtained with FDG-PET is related to glucose metabolism in tumor cells, stromal cells or both and to what extent in which tumor entity.

## Fibroblast activation protein as an important marker of CAFs

Due to their heterogeneity of origin and, therefore, the heterogeneity of the expression pattern, it is difficult to use a unique marker which can be used for the identification of all CAFs (Averya et al. 2018; Cortez et al. 2014; Kilvaer et al. 2018). Kilvaer et al. found in an immunohistochemistry analysis of micro arrays in 536 patients with NSCLC that the fibroblast and stromal markers PDGFRα, PDGFRβ, FAP-1 and vimentin showed only weak correlations. In contrast, αSMA did not correlate with any of the other markers. Therefore, the presence of phenotypically different subsets of CAFs may differ between tumor regions due to heterogeneity of origin (Kilvaer et al. 2018).

However, there is at least one marker which is overexpressed in the stroma of many tumor entities and potentionally usefull for imaging and therapy: the fibroblast activation protein. FAP is a type II membrane bound glycoprotein belonging to the dipeptidyl peptidase 4 (DPP4) family. This family consists of several members: DPP4, quiescent cell proline dipeptidase (QPP), FAP, prolyl oligopeptidase (POP), DPP8, DPP) and the inactive members DPP6 and DPP10 (Juillerat-Jeanneret et al. 2017). DPP4 and FAP are membrane–bound enzymes and show a 52% identity at the protein level (Jacob et al. 2012). In contrast to DPP4, having only exopeptidase activity, FAP shows both dipeptidyl peptidase and endopeptidase activity. (Hamson et al. 2014). Identified substrates for the endopeptidase activity so far are gelatin, denatured type I collagen, α-antitrypsin and several neuropeptides such as neuropeptide Y, peptide YY, B-type natriuretic peptide and substance P (Hamson et al. 2014; Keane et al. 2011; Kelly et al. 2012; Lee et al. 2004; Park et al. 1999). The enzyme is known to have a role in normal developmental processes during embryogenesis and in tissue modelling (Niedermeyer et al. 2000; Niedermeyer et al. 2001; Brown et al. 1996; Jacob et al. 2012). It is not or only at insignificant levels expressed on adult normal tissues. Examples are uterus, cervix, placenta, breast and skin, which show a low to moderate expression as compared to tumors (Rettig et al. 1994; Dolznig et al. 2005). However, high expression occurs in wound healing, inflammation such as arthritis, artherosclerotic plaques, fibrosis (Egger et al. 2017, Uitte de Willige et al. 2013) as well as in ischemic heart tissue after myocardial infarction (Tillmanns et al. 2015; Nagaraju et al. 2017) and in more than 90% of epithelial carcinomas (Bauer et al. 2006, Brokopp et al. 2011, Levy et al. 2002, Levy et al. 1999, Mentlein et al. 2011, Park et al. 1999, Rettig et al. 1988, Scanlan et al. 1994, Wang et al. 2005,).

FAP has a total of 760 amino acids with an intracellular domain of only 6 amino acids and a transmembrane domain with 20 amino acids. The largest part is the extracellular domain which consists of a β-propeller domain and a catalytic domain (Kelly 2005; Wang et al. 2008; Jacob et al. 2012). In the catalytic domain serin (S624), aspartate (D702) and histidine (H734) form a triad, which classifies FAP as a serine protease (Aertgeerts et al. 2005; Goldstein et al. 1997; Kelly et al. 2012; Piniero-Sanchez et al. 1997; Scanlan et al. 1994). Serine 624 is involved in dipeptidyl peptidase as well as in endopeptidase activity. Furthermore, the catalytic site consists of a Gly-X-Ser-X-Gly sequence. FAP is produced as a 97 kDa subunit which needs dimerization to be enzymatically active. Usually this occurs as homodimer, but also heterodimers with DPP4 and β1 integrins have been found (Artym et al. 2002; Rettig et al. 1993). FAP expression seems to be regulated via different transcription factors such as early growth response (EGR-1), HOXA4 and E2F1 as shown by FAP promoter analysis (Zhang et al. 2010).

Shedding may occur leading to soluble forms which have been detected in the plasma (Niedermeyer et al. 1997; Lee et al. 2004; Wild et al. 2010; Piniero-Sanchez et al. 1997; Mueller et al. 1999).

## Therapies targeting CAFs

Since CAFs play a critical role in tumor growth, migration and progression, are genetically more stable than cancer cells and less susceptible to the development of therapy resistance, they represent excellent target cells for antitumor therapy. FAP is broadly expressed in the microenvironment of a variety of tumors and thus allows targeting of different tumor entities including pancreas, breast and lung cancer, which account for a large part of the entirety of solid tumors. Therefore, several approaches have been used to treat tumors by targeting FAP: immunoconjugates (Ostermann et al. 2008), CAR T cells (Wang et al. 2014; Lo et al. 2015), tumor immunotherapy (Lee et al. 2005), vaccines (Loeffler et al. 2006), peptide drug complexes (Brennen et al. 2012a, 2012b; Chen et al. 2017; LeBeau et al. 2009; Wang et al. 2017), FAP inhibitors (Teichgräber et al. 2015) and antibodies (Hofheinz et al. 2003; Scott et al. 2003; Welt et al. 1994; Wüst et al. 2001).

Preclinical studies with anti-FAP antibodies revealed promising results (Loeffler et al. 2006; Ostermann et al. 2008). One study used an oral DNA vaccine targeting fibroblast activation protein for specific elimination of FAP-expressing fibroblasts in multi-drug resistant colon and breast cancer models (Loeffler et al. 2006). This strategy led to a CD8+ T cell-mediated killing the fibroblasts, suppression of primary tumor cell growth as well as metastasis. In addition, effects on the microenvironment were observed such as a decrease in collagen type I expression. Chemotherapeutic drugs showed an up to 70% higher uptake in these tumors with longer lifespan in these animals. Similarly, treatment with an antibody-maytansinoid conjugate (mAb FAP5-DM1) led to a long-lasting inhibition of tumor growth and to complete regressions in a variety of different xenograft models. This was related to mitotic arrest and apoptosis in malignant epithelial cells and disruption of fibroblastic and vascular structures (Ostermann et al. 2008). However, these positive effects could not be seen in clinical application (Hofheinz et al. 2003). In this open-label, uncontrolled, multicentre study sibrotuzumab (BIBH 1) was given to 17 patients with metastatic colorectal cancer. After 8 infusions of sibrotuzumab tumor progression was seen in 15 patients and stable disease in two patients which developed progression after 1 und 6 additional infusions (Hofheinz et al. 2003).

If CAFs are important regulators of the tumor microenvironment with direct and indirect influences on growth, migration, immune reactions and also resistance to conventional therapy, then a selective depletion may result in detrimental affects on the tumor. This depletion may be obtained by targeting of fibroblasts specific prodrugs, CAR T cells, antibodies or specific ligands labelled with α-emitting radionuclides. However, questions about the safety of this approach may arise. These are related to the heterogeneity of the different fibroblast subpopulations in the tumor. As shown by Averya et al. (2018) this heterogeneity is the result of a convergence of extracellular matrix composition, tissue elasticity, and transforming growth factor β (TGF-β) signaling. In their study FAP-positive fibroblasts and αSMA-positive myofibroblasts had distinct gene expression signatures and and accordingly a different functionality. Although FAP is expressed on the majority of activated fibroblasts in the tumor microenvironment, only a subset of these cells co-express αSMA (Kilvaer et al. 2018; Lo et al. 2015; Pure and Blomberg 2018). Specific ablation of αSMA-postive myofibroblasts in mouse models of pancreatic cancer led to suppression of the anti-tumor immunity, enhancement of hypoxia and endothelial to mesenchymal transition, and reduction of survival (Ozdemir et al. 2014). A further finding of the experiments of Ozdemir et al. was that the depletion of the αSMA-positive fibroblasts had no effect on the amount of the FAP-positive fibroblasts with only a modest and selective effect on the extracellular matrix. In contrast, the specific depletion of FAP-positive fibroblasts either with genetic approaches (Feig et al. 2013; Arnold et al. 2014; Kraman et al. 2010) and immunologic methods (Lee et al. 2005; Loeffler et al. 2006; Kakarla et al. 2013; Ostermann et al. 2008; Schuberth et al. 2013; Tran et al. 2013; Wang et al. 2014) resulted in an inhibition of tumor growth. This suggests that αSMA and FAP fibroblasts may influence the tumor microenvironment differently. Indeed, some of the available studies provided direct evidence that the depletion of FAP-positive CAFs leads to an enhanced anti-tumor immunity (Feig et al. 2013; Arnold et al. 2014; Kraman et al. 2010; Lo et al. 2015; Pure & Lo 2016; Wang et al. 2014).

FAP-targeting strategies were shown to be more effective when combined with other treatment modalities such as chemotherapy, vaccination or antibodies (Brünker et al. 2016, Fang et al. 2016a, 2016b, Fang et al. 2016a, 2016b, Huang et al. 2015, Xia et al. 2017, Zhang et al. 2016). This may be an approach to address the lack of efficacy observed with sibrotuzumab (Hofheinz et al. 2003).

Since FAP is an enzyme, work on FAP also focussed on either FAP substrates or FAP inhibitors. These could be used either to design prodrugs which are activated specifically at the tumor site by the FAP activity or selective enzyme inhibitors. Activable prodrugs have been used using FAP substrates coupled to cytotoxic drugs such as melittin, doxorubicin, thapsigargin (Akinboye et al. 2016; Brennen et al. 2012a, 2012b; Huang et al. 2011a, 2011b; Ke et al. 2017; LeBeau et al. 2009), but so far no clinical success has been reported.

Enyzme inhibitors were synthesized targeting the NH_2_-Xaa-Pro motif leading to Pro-boroPro, Ala-boroPro, and Val-boroPro compounds (Connolly et al. 2008; Flentke et al. 1991; Poplawski et al. 2013). The resulting molecules were effective against most post-prolyl peptidases and, therefore, had broad specificity. As an example, Val-boro-Pro (PT-100, talabostat) proved to be a promising compound in preclinical studies (Egger et al. 2017, Huang et al. 2011a, 2011b, Li et al. 2016, Pennisi et al. 2009). However, clinical trials with talabostat revealed only minimal effects, even when combined with chemotherapy (Narra et al. 2007; Eager et al. 2009a, 2009b).

In an attempt to develop FAP-specific inhibtors, Jansen et al. (2013 and 2014) examined a variety of struturally related small molecules with some of them being highly specific for FAP. These molecules could be used as lead structures for new radiopharmaceuticals.

## Radionuclide based imaging and therapy

FAP imaging has been done with antibodies and an inhibitor molecule (Laverman et al. 2015; Meletta et al. 2015; Tanswell et al. 2001; van der Geest et al. 2017). For the detection of atherosclerotic plaques the boronic acid based FAP inhibitor MIP-1232 was used in a preclinical study (Meletta et al. 2015; Zimmermann et al. 2010). Iodine-labelled MIP-1232 (Zimmermann et al. 2010) showed high accumulation in FAP-positive SK-Mel-187 xenografts. However, the binding of the compound to endarterectomized tissues was similar in artherosclerotic plaques and normal arteries, indicating that atherosclerosis imaging using this compound may be difficult (Meletta et al. 2015). In contrast imaging of rheumatoid arthritis has been performed in animal models using the antibody 28H1 labeled with ^111^In, ^89^Zr or ^99m^Tc revealing a high tracer uptake in inflamed joints. In this study the tracer accumulation was correlated to the arthritis score (Laverman et al. 2015; van der Geest et al. 2017).

Furthermore, the anti-FAP antibody sibrotuzumab labeled with ^131^I was applied for the treatment of patients with metastasized FAP-positive carcinomas (Scott et al. 2003; Welt et al. 1994). ^131^I-sibrotuzumab revealed a slow elimination in the liver, the spleen and other normal organs which was consistent with a slow blood pool clearance. However, considerable tracer accumulation was measured in metastatic lesions larger than 1.5 cm in all patients, which occurred usually at day 2 after administration (Scott et al. 2003). The detection rate could be improved using the SPECT technique detecting lesions down to 1 cm in diameter (Welt et al. 1994). Consistent with the general pharmacokinetics of antibodies the optimal time for imaging was found to be 3 to 5 days post injectionem.

In addition to the slow clearance of sibrotuzumab, leading to a high background signal, another shortcoming of this approach was the use of ^131^I for either planar imaging or SPECT. The high energy gamma emission requires the use of high energy collimators and thick crystal detectors with a negative impact on image resolution. Together this results in a limited sensitivity for the detection of small lesions. This can be encountered by applying radiolabelled small molecules such as MIP-1232 or other FAP inhibitors. Using this approach, the limiting factor for the detection of tumor lesions is the number of FAP-positive cells in the tumor micrenvironment i.e. the percentage of stromal content, and/or the number of FAP molecules per fibroblast which may be determined by the surrounding cells (Loktev et al. 2018). Since tumor lesions exceeding a size of 1 to 2 mm require the formation of a supporting stroma (Davidson et al. 1997), visualization of small lesions in the range of 3–5 mm should be possible using these molecules (Loktev et al. 2018).

Based on a small molecule enzyme FAP inhibitor (FAPI) with high affinity designed by Jansen et al. (2013), we first developed the radiotracers FAPI-01 and FAPI-02 which showed specific binding to human and murine FAP with a rapid and almost complete internalization without addressing the closely related protein DPP4/CD26 (Loktev et al. 2018). FAPI-01 proved to be suboptimal due to enzymatic deiodination with efflux of free iodine and consequently a lower intracellular radioactivity after longer incubation intervals.

In contrast, FAPI-02 which is a DOTA-linked compound, showed better pharmacokinetic and biochemical properties. The compound was eliminated much slower than FAPI-01, with an approximately 10-fold higher retention after 24 h. Furthermore, a rapid internalization into FAP expressing cells was seen with a high tumor uptake both tumor xenografts and patients with metastasized epithelial carcinomas (Loktev et al. 2018, Giesel et al. 2019a, 2019b).

In 80 patients with 28 different tumor entities (54 primary tumors and 229 metastases) a quantitative analysis of tracer uptake was done. The highest average SUVmax (> 12) was found in sarcoma, esophageal, breast, cholangiocarcinoma and lung cancer, the lowest uptake (average SUVmax < 6) was observed in pheochromocytoma, renal cell, differentiated thyroid, adenoid-cystic and gastric cancer and an intermediate SUVmax (SUV 6–12) was seen in patients with hepatocellular, colorectal, head-neck, ovarian, pancreatic and prostate cancer. However, the SUV showed a considerable variation across and within all tumor entities. Since the background in muscle and blood-pool was very low (SUVmax < 2), the tumor-to-background contrast ratios were > 3-fold in the intermediate and > 6-fold in the high uptake group. (Kratochwil et al. 2019).

The internalization into FAP expressing cells was confirmed by confocal microscopy with a fluorescence-labeled FAPI-02 molecule (Loktev et al. 2018). In contrast to the FAP-antibody F19, which is known to have a high affinity without being internalized, FAPI-02 revealed an almost complete internalization after 1 h incubation. The mechanism of internalization after FAP binding has been elaborated with FAP antibody fragments (Fabs) in SK-Mel-187 cells showing also a rapid and almost complete internalization (Fischer et al. 2012). Furthermore, a colocalization of the antibody fragments with an early endosome marker was observed after 20 min and with a late endosome and lysosome marker after 40 min. The internalization of the anti-FAP-Fab/FAP complex could be suppressed by an inhibitor for dynamin dependent endocytosis (Fischer et al. 2012).

Since there is a very low accumulation in normal tissues and a rapid clearance from the circulation, a high-contrast is obtained for PET imaging. Furthermore, FAPI-02 is quickly eliminated from the organism by renal clearance without being retained in the renal parenchyma which is favorable for a possible therapeutic application. In contrast to ^18^F-FDG, which shows a high uptake in cells with a high need for glucose such as inflammatory tissue or the brain, FAPI-02 gets selectively enriched in tissues where its target protein is expressed. The fact that there is no or a very low FAPI-02 uptake in all normal organs, especially the brain and the liver, opens new possibilities for the detection of malignant lesions in these regions based on the high contrast images obtained with that compound (Loktev et al. 2018, Giesel et al. 2018).

In order to optimize the uptake and tracer retention in the tumor, a series of compounds based on FAPI-02 was developed (Lindner et al. 2018). Improvement of tracer properties was obtained by utilization of 4,4-difluoroproline, which has been described previously by Jansen et al. (2013, 2014). This substitution led to a 3-fold reduction of the EC_50_ value for FAPI-04, one of the compounds tested, and a shift of the difference in affinity for the related enzymes FAP and DPP4/CD26 with FAP/DPP4 binding ratios of 45 and 750 for FAPI-02 and FAPI-04. In vitro efflux experiments revealed a significantly slower washout of FAPI-04. Small animal PET imaging showed a higher tumor uptake, longer dwell time and no significant increase of activity in normal organs. The analysis of stability in human serum revealed no degradation during 24 h (Lindner et al. 2018). A comparison of the different molecules revealed that some modifications are necessary to improve tumor retention: the heterocyclic segment, the position of the linker at the quinolone moiety and the bond between DOTA and the propylamine as opposed to the piperazine bond.

In order to enhance cellular retention by intracellular transport into the nucleus, FAPI-10 was designed by adding a nuclear localization signal. However, although the target binding was significantly higher compared to FAPI-02 in vitro, the tumor retention time in vivo was not improved and the presence of the peptide sequence led to a high accumulation in the the kidneys disqualifying this compound for clinical translation.

A preliminary dosimetry estimate for FAPI-02 and FAPI-04 was done in two patients examined at 0.2 h, 1 h and 3 h after tracer injection revealing a dose of 1.4–1.8 mSv/100 MB which corresponds to an equivalent dose of approximately 3–4 mSv for an injected activity of 200 MBq (Giesel et al. 2018). Therefore, the effective dose of a ^68^Ga FAPI PET is comparable to other ^68^Ga-based tracers such as ^68^Ga-DOTATOC/DOTATATE (2.1 mSv/100 MBq) or ^68^Ga-PSMA-11 (1.6–2.4 mSv/100 MBq) and also to the dose abtained after administration of with FDG (2 mSv/100 MBq).

**Fig. 1:**
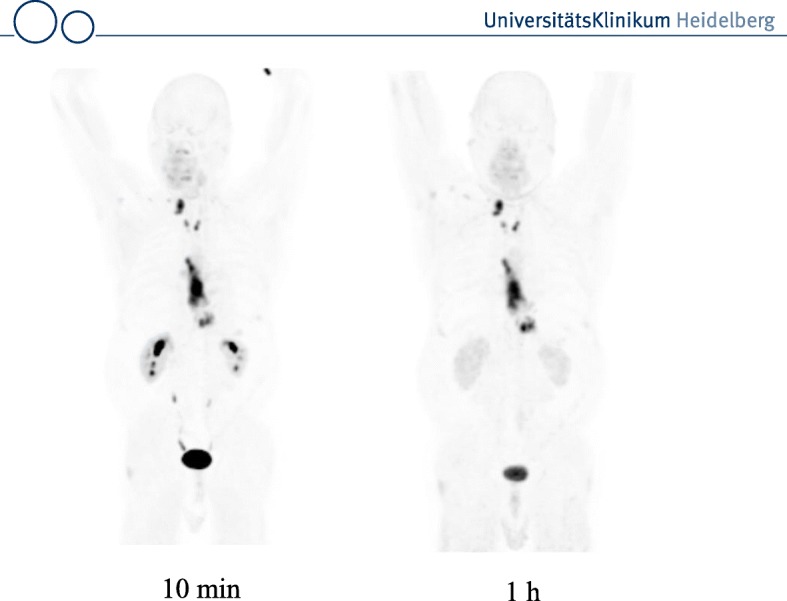
Patient with esophageal cancer and lymph node metastases. The MIPs at 10 min and 1 h p.i. show tracer accumulation in the tumor and lymph node metastases. Radioactivity is also seen in the renal pelvis, the ureter (at 10 mintes) and the bladder. In contrast, the renal parechnyma shows a very low signal

In patients both tracers showed a fast renal clearance resulting in a low uptake in the normal organs. Usually radioactivity was seen only in the renal pelvis and the bladder, with no accumulation in the renal parenchyma. Furthermore, only minimal changes in background were observed between 10 min and 3 h p.i.. Visualization of metastases with high contrast was obtained even at 10 min after tracer administration. This is illustrated by Fig. 1 which shows MIPs of patient with esophageal cancer at 10 min and 1 h p.i.. For FAPI-02 tumor uptake decreased by 75% from 1 h to 3 h. In contrast, FAPI-04 showed a prolonged tumor retention with 25% washout. At 1 h both FAPI-tracers performed equally with respect to tumor-to-background ratios.

**Fig. 2:**
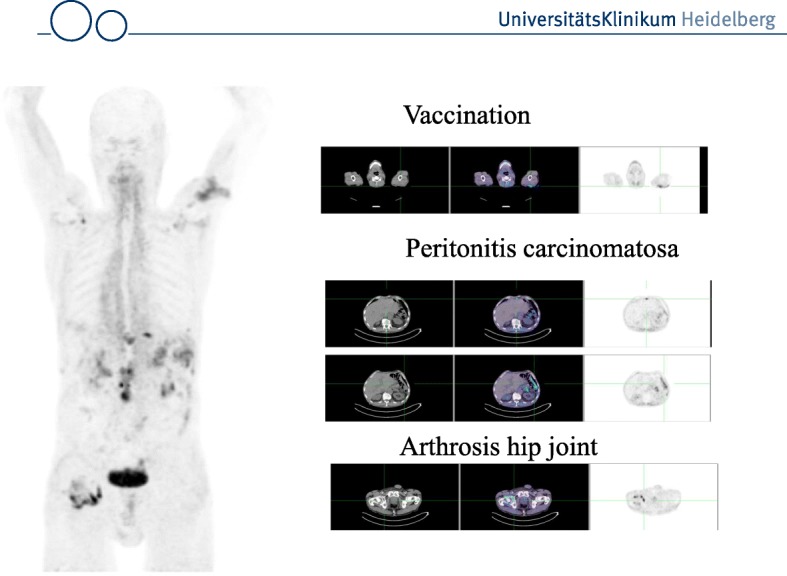
MIP and transaxial slices (CT, fusion image and PET) of a patient with gastric cancer and peritonitis carcinomatosa: tracer uptake is seen in the left shoulder, the abdomen, and the right hip joint. The accumulation in the left shoulder resulted from a 6 month enduring vaccination with peptides leading to a chronic inflammation. The uptake in the right hip joint correspond to an activated arthritis, whereas the multiple lesions in the abdomen are caused by the peritonitis carcinomatosa

**Fig. 3:**
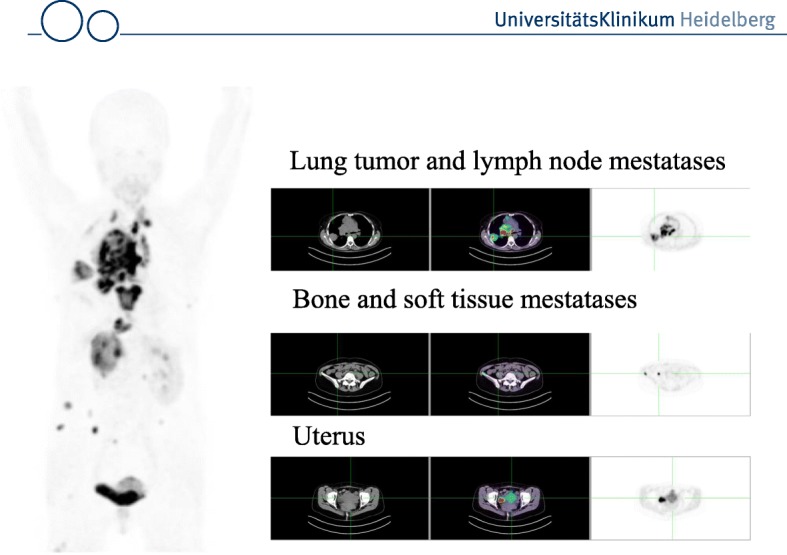
MIP and transaxial slices (CT, fusion image and PET) of a patient with non small cell lung cancer. Tracer uptake occurs in the primary tumor, mediastinal lymph node metastases, bone metastases and a soft tissue metastasis. Physiological uptake is seen in the uterus

A comparison to FDG in 6 patients revealed that the tumor uptake was almost equal (average SUV_max_-FDG 7.41; SUV_max_-FAPI-2 7.37; n.s.). However, the background uptake in brain (11.01 vs 0.32), liver (2.77 vs 1.69) and oral/pharyngeal mucosa (4.88 vs 2.57) was significantly lower with FAPI. This seems to be a promising feature of the FAPI tracers with respect to the detection of brain or liver metastases, liver tumors or head-and-neck tumors. As expected accumulation of FAPIs was observed not only in tumors, but also at sites with tissue remodeling such as chronic inflammation after vaccination, activated arthrosis (Fig. 2) and physiologically in the uterus of a 53 year old patient (Fig. 3). Since the presence of activated fibroblasts in the injured myocardium may predict the cardiac remodelling after myocardial infarction (MI), ^68^Ga-FAPI-04 was used in a rat model of MI (Varasteh et al. 2019). Tracer uptake in the injured myocardium peaked at day 6 after coronary ligation in the area of the MI territory. This finding was confirmed by immunofluorescence which revealed the presence of FAP-positive myofibroblasts in the infarct area. This indicates that the imaging of activated fibroblasts may have a diagnostic and prognostic value in the setting of the management of patients with MI.

A high FAPI uptake was found in pancreatic cancer, esophageal cancer, NSCLC, head and neck cancer and colon cancer, whereas a patient with dedifferentiated thyroid cancer with high FDG uptake in the tumor lesions showed a low FAPI uptake or was FAPI-negative (Giesel et al. 2018). However, advantages of FAPI imaging over FDG could be the independency of blood sugar levels, no need for resting and the possibility of early imaging even at 10 min p.i. which may be used to reduce the waiting time or the amount of activity given to the patient. Furthermore, the FAPI ligands are chelator-based containing DOTA which may be used for therapeutic applications. In order to fit the physical half-life of the radionuclide used for therapy to the retention in the tumor, ^90^Y-90 with a half-life of 64 h was chosen for a proof of principle approach in a final stage breast cancer patient with bone metastases which was treated with 2.9 GBq ^90^Y-FAPI-04. Visualization of the metastases in Bremsstrahlung images was possible even at 24 h after tracer administration (Lindner et al. 2018). This was associated with a significant reduction of opioids given as pain medication. Furthermore, no side effects were observed, especially no therapy related hematotoxicity.

**Fig. 4:**
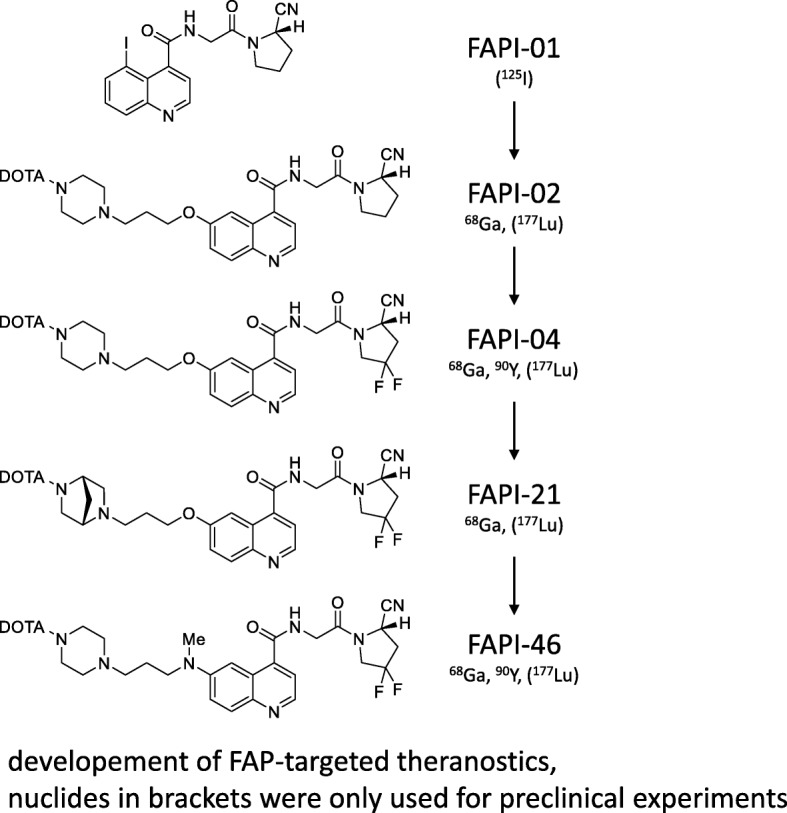
Development of FAP-targeted theranostics. Chemical structures are shown for selected compounds which were investigated in detail preclinically and/or clinically. Radionuclides in brackets were only used for preclinical experiments

Further modifications were done with a focus on prolonged tumor retention while retaining the imaging contrast obtained with FAPI-02 and FAPI-04. This was done either by alteration of the lipophilicity by variations of the linker region mainly by bicyclic analogues of the original piperazine moiety or by modification of the chemistry used for DOTA/linker-attachment at the quinoline moiety (Loktev et al. 2019). An overview of the most important FAPI variants is given in Fig. 4. Compared to FAPI-04, 11 out of 15 FAPI derivatives showed improved FAP binding in cell culure experiments. Seven compounds showed an increased tumor uptake and high tumor-to-organ ratios in small animal PET imaging and biodistribution studies. FAPI-21 and FAPI-46 revealed substantially improved ratios of tumor to blood, liver, muscle, and intestinal uptake and FAP-specific binding in vivo which was verified in competition experiments with a complete blocking of tumor accumulation after addition of unlabeled compound. A first diagnostic application in cancer patients revealed a high intratumoral uptake of both radiotracers which occured early at ten minutes p.i.. However, FAPI-21 showed a higher uptake in oral mucosa, salivary glands and thyroid which was not the case for FAPI-46.

In human tumors, the origin, number and distribution of FAP-expressing CAFs as well as the number of FAP molecules per cell may differ. This may result in different pharmacokinetic profiles of the radiotracers in different tumor entities. Conseqently, in a first small series of patients a different kinetics from 1 h to 3 h p.i. was seen in different tumor entities: a constant intracellular activity in colorectal, ovarian, oropharynx and pancreatic carcinoma, a continuous decrease in breast carcinoma and an increasing tracer accumulation in one patient with carcinoma of unknown primary (Loktev et al. in revision). As mentioned above, this may be due to the heterogeneous origin of CAFs, which may develop from resident fibroblasts, bone marrow derived mesenchymal stem cells, endothelial cells, epithelial cells and even adipocytes. This difference in origin is liely to result in different proteomes with a considerable expression of CAF markers such as FAP, αSMA or PDGFRβ. However, this observation has to be evaluated in a larger number of patients. From these studies we may expect important information with respect to the indication of a FAPI-based endoradiotherapy: tumors with a longer retention may respond better than tumors with a fast elimination of the radiopharmaceutical.

## Conclusion

In conclusion, FAPIs are promising tracers for diagnostic applications not only for tumors showing a desmoplastic reaction, but also for non-malignant diseases with tissue remodeling such as myocardial infarction, sarcoidosis, chronic as opposed to acute inflammation, lung, liver and kidney fibrosis, rheumatoid arthritis and artherosclerosis. For a therapeutic application the physical half-life of the radionuclide has to be adjusted to the retention time: Radionuclides with shorter half lives seem to be preferable than radionuclides with a longer half life. This would be in favor of ^188^Re, ^153^Sm, ^213^Bi or ^212^Pb. Furthermore, a combination of an α and a β emitter seems promising by for a simultaneous irradiation of CAFs and the surrounding tumor cells. A further promising aspect of a therapeutic application is the combination with radiation therapy by increasing the local dose or enabling a reduction of the externally delivered radiation leading to a decrease in side effects. Finally, since FAP-expressing CAFs are known to be immunosuppressive, a combination with immunotherapy may lead to a synergizing effect.

## Data Availability

Not applicable.

## Abbreviations

CAFs: Cancer-associated fibroblasts
CCL: Chemokine (C-C motif) ligands
DPP4: Dipeptidyl peptidase 4
EGR-1: Early growth response 1
FAP: Fibroblast activation protein
FGF: Fibroblast growth factor 1 and 2,
FSP1: Fibroblast stimulating protein 1
HGF: Hepatocyte growth factor
MCTs: Monocarboxylate transporters
PDGFR β: Platelet derived growth factor β
POP: Prolyl oligopeptidase
QPP: Quiescent cell proline dipeptidase
SDF1: Stromal cell derived factor 1
TGFβ: Transforming growth factor β
αSMA: α smooth muscle actin
